# Exploring “psychic transparency” during pregnancy: a mixed-methods approach

**DOI:** 10.1186/s12905-016-0332-4

**Published:** 2016-08-12

**Authors:** Cécile Oriol, Sylvie Tordjman, Jacques Dayan, Patrice Poulain, Ouriel Rosenblum, Bruno Falissard, Asha Dindoyal, Florian Naudet

**Affiliations:** 1 Pôle Hospitalo-Universitaire de Psychiatrie de l’Enfant et de l’Adolescent (PHUPEA), Université de Rennes 1, Centre Hospitalier Guillaume Régnier, Rennes, France; 2 Laboratoire de la Psychologie de la Perception, CNRS UMR 8158, Université Paris-Descartes, Paris, France; 3 Inserm-EPHE-Université de Caen/Basse-Normandie, Unité U923, GIP Cyceron, CHU Côte de Nacre, Caen, France; 4 Pôle obstétrique, gynécologie, biologie de la reproduction, chirurgie plastique et reconstructrice, hôpital SUD, CHU de Rennes, Rennes, France; 5 Service de Psychiatrie de l’enfant, Pitié-Salpêtrière, Paris, France; 6 INSERM U669, Paris, France; 7 Centre d’Investigation Clinique, INSERM 0203, Unité de Pharmacologie Clinique Unit, Universitary Hospital and University of Rennes 1, Rennes, France; 8 Centre d’Investigation Clinique INSERM 1414, Unité de Pharmacologie Clinique, Hôpital de Pontchaillou, 2 rue Henri le Guilloux, 35033 Rennes cedex 9, France

**Keywords:** Psychic transparency, Pregnancy, Dreams, Psychodynamic, Mixed methods

## Abstract

**Background:**

Psychic transparency is described as a psychic crisis occurring during pregnancy. The objective was to test if it was clinically detectable.

**Methods:**

Seven primiparous and seven nulliparous subjects were recorded during 5 min of spontaneous speech about their dreams. 25 raters from five groups (psychoanalysts, psychiatrists, general practitioners, pregnant women and medical students) listened to the audiotapes. They were asked to rate the probability of the women being pregnant or not. Their ability to discriminate the primiparous women was tested. The probability of being identified correctly or not was calculated for each woman. A qualitative analysis of the speech samples was performed.

**Results:**

No group of rater was able to correctly classify pregnant and non-pregnant women. However, the raters’ choices were not completely random. The wish to be pregnant or to have a baby could be linked to a primiparous classification whereas job priorities could be linked to a nulliparous classification.

**Conclusions:**

It was not possible to detect Psychic transparency in this study. The wish for a child might be easier to identify. In addition, the raters’ choices seemed to be connected to social representations of motherhood.

## Background

### Noteworthiness of the problem

Maternal health is an important determinant in women’s health [1] which is not restricted to the technical management of pregnancy. From a holistic point of view, maternity care encompasses physiological and psychological aspects. Prenatal and postnatal care alike relies on a clinical encounter and requires sensitivity to the dynamics of the relationship. Psychoanalysts describe a particular dynamic during pregnancy involving a psychological crisis and a process of maturation arising from the experience of a new developmental phase, parenthood [2]. Several authors have described a specific psychic state during pregnancy. Among them, Donald Winnicott developed the concept of “primary maternal preoccupation” [3] which refers to a new mind-set which emerges during pregnancy and may last for few weeks after delivery. During this time, the future mother goes through a “normal illness” which enables her to feel connected to her baby and to understand his needs better in order to create a good environment for the future infant. In addition, Daniel Stern refers to a specific mental organization during pregnancy through the concept of the motherhood constellation [4]. When expecting a baby, the mother’s self sense becomes largely organized around the presence of the fetus, its well-being and their mutual connection. In line with this, Joan Raphael-Leff set out what she called “the Healthy Maternal Ambivalence” and the importance of considering pregnant women with their subjectivity, giving the right dimension to the complex interpersonal dynamics between a mother and her future baby [5]. In line with these authors, Monique Bydlowski was inspired by her specific practice (psychiatrist, psychoanalyst, as well as working in obstetrics) to develop her own concept called “psychic transparency” [6, 7]. This term refers to a particular psychological state in which the mental functioning of the mother seems to be easier to read and to capture than it usually is [6] with a decrease in the censorship of thoughts, resulting in a more explicit expression of desires, conflicts and impulses, and in relational regressions [7]. Because of the presence of the fetus, the disinvestment of the childhood Oedipal objects of the future mother may be one of the mechanisms explaining this decrease in the censorship of thoughts. Indeed, the fetus can be considered as the embodiment of the Oedipal desire. Psychic transparency is linked to the worries arising from the unusual thoughts of the future mother about the integrity of the baby or the fear of harming him. This could be explained by the porosity of the preconscious area to primary processes as a result of the partial decrease in the censorship of thoughts. This state is described as common and easy to identify even for a non-psychoanalyst observer. Surprisingly, it has been reported that evocation of the unborn child is not as frequent as might be thought in the spontaneous discourse of pregnant women experiencing a peaceful pregnancy. This may reflect an inner happiness which does not need to be shared. “When everything is going well, nothing needs to be said” [6].

### Theoretical framework

Like many concepts described in the field of psychodynamic psychopathology, the notion of “psychic transparency” originated from a qualitative approach based on the clinical case study method. This method provides detailed information, generates working hypotheses, but presents problems in terms of generalization, and is prone to a researcher bias (one’s own subjective feelings can influence the case study) [8–10]. On the other hand, the holistic and phenomenological dimensions of “psychic transparency” make it difficult for it to be apprehended with a conventional quantitative approach using, for example, psychometric scales or questionnaires. An experimental design based on a generalization of Fisher’s «Lady-Tasting-Tea» procedure [11] has been proposed to link qualitative and quantitative approaches [12, 13] with the ambition of “articulating science and humanism in the service of the wholeness of the person who consults” [14].

### The suitability of the issues to mixed-methods design

If there is a “psychic transparency” during pregnancy, phenomenological differences should allow different observers to differentiate the status (pregnant or not) of women. Additionally, we hypothesized that personal psychodynamic experience would enhance this ability. Thus, our principal objective was to quantitatively explore psychoanalysts’ ability to discriminate pregnant from non-pregnant women on the basis of a sample of free-association speech samples. Our secondary objectives were 1/ to quantitatively explore the same ability among non-psychoanalysts, 2/ to assess qualitative differences between pregnant and non-pregnant women, and 3/ to explore the evaluators’ representations of pregnancy.

## Methods

### Subjects

Women were recruited from the University Department of Obstetrics and Gynecology in Rennes (France). They were eligible for inclusion if they 1/ were aged from 19 to 34 years, 2/ had been in a relationship with a partner for over a year, 3/ were free from history of miscarriage, 4/ were able to understand the protocol and to speak French fluently and 5/ gave their consent. They were not included if they had 1/ psychiatric comorbidity assessed by the Mini International Neuropsychiatric Interview [15], 2/ significant medical illness, 3/ guardianship and 4/ a partner with children from a previous relationship.

The pregnant women were primiparous (G1P0), in the third trimester of pregnancy. They were not included in case of risk-prone or pathological pregnancy. The non-pregnant women were nulliparous (G0P0) and were matched for age, educational level and lifestyle (rural or urban).

### Procedure

All the eligible women were asked for a 30-minute interview. The first part of the interview was used 1/ to check selection criteria, 2/ to collect socio-demographic and anamnestic data and, 3/ to create an empathetic and caring atmosphere liable to establish a working alliance. Then the women were audiotaped for 5 min of spontaneous speech. They were asked not to talk about their pregnancy and were excluded in case of explicit reference to it. The instructions were as follows (translated into English here to provide information on the content): “Take roughly 5 min to talk about yourself. For instance, you could talk about the value you attach to your dreams, about their possible meaning and about their importance in the morning. How much room do you give them in your everyday life? You could also talk about their strangeness and their impact in your life, for example, the feeling of “déjà vu”, or the feeling of being in a waking dream. You could also talk about the nature of your relationship with yourself: are you interested in your inner life, or are you more attracted by external reality? Finally, and this is the most important thing, try to speak freely, saying whatever comes to mind and following your thoughts freely.” These instructions were adapted from previous research [12], with a special emphasis on dreams because Freud’s assertion that they are the “highway to knowledge of the unconscious activities of the mind” [16] seems particularly true for pregnant women [17, 18]. Many articles have focused on the potential function of dreams during pregnancy [17] and on the potentially specific themes of these dreams [19]. Most studies chose to focus on the explicit content of the dream [20] whereas we chose to pay attention to the implicit content. At the end of the interview, subjects were asked to complete the State-Trait Anxiety Inventory (STAI) [21].

### Outcomes and raters

The 5-minute audiotapes were presented blind to different evaluators (with a different randomized order for each evaluator). The primary outcome was the psychoanalysts’ ability to differentiate the women (pregnant or non-pregnant). Psychoanalysts were recruited from psychiatry or medical psychology teams. They were all receiving and practicing psychodynamic therapy themselves. All of them were in active psychodynamic supervision. As “psychic transparency” is described as something that is easy to identify, even for a non-psychoanalyst observer, 4 other sets of evaluators were included. Their ability to differentiate the women was a part of our secondary outcomes: 1/ psychiatrists without experience of psychoanalysis, 2/ general practitioners without experience of structured psychotherapy, 3/ medical students who are inexperienced professionals and 4/ pregnant women to explore the possible influence of an “internal echo”. All these evaluators were asked to rate the probability of the woman being pregnant or not (response options: surely pregnant, may be pregnant, unlikely to be pregnant, certainly not pregnant) given that half of the women were pregnant and the other half were not.

A qualitative analysis of the audiotapes was performed to apprehend and to compare the experiences of pregnant and non-pregnant women, in a phenomenological approach.

To explore the evaluators’ representations of pregnancy, a final secondary outcome was the probability for each woman of being classified as pregnant or not by all the raters.

### Quantitative analysis

For each audiotape, and for each rater in each group, a score of +2 or +1 or −1 or −2 was added to a global score on the basis of their answers (Table 1).

**Table 1:** Modification of the global score from each audiotape rating

Rating	For a pregnant woman	For a non-pregnant woman
Surely pregnant	+2	− 2
Likely to be pregnant	+1	− 1
Unlikely to be pregnant	− 1	+1
Certainly not pregnant	− 2	+2

A global score was thus obtained from the answers given by all the raters to all the audiotapes. A permutation test was used to compare this global score to 0 (under the null hypothesis that the evaluators cannot differentiate the pregnant from the non-pregnant women). Indeed, under the null hypothesis that recordings by pregnant women and non-pregnant women are indistinguishable, all permutations of scores obtained for each record should be equi-probable. A *p*-value was therefore estimated as the proportion of permutations of the n recordings for which the total score was higher than or equal to the total score obtained in the experiment [13]. This analysis was carried out in each evaluator group. To find out whether a particular woman was classified pregnant or non-pregnant more often than would have been expected by chance, a global score was also calculated for each woman and the bilateral probability of obtaining this score under the null-hypothesis was computed. Other quantitative data were compared between groups using non-parametric tests. All statistical analysis were performed using R software [22] and the *p*-value for statistical significance was defined as *p* < 0.05.

### Sample size calculation

We hypothesized a sensitivity and specificity of 0.8 for the psychoanalysts’ ability to identify pregnant women correctly. Following recommendations by Falissard et al. [13], with a power of 0.96, seven patients per group were necessary, and five evaluators. Under the same hypotheses, five raters per group were recruited to test the secondary outcomes.

### Qualitative analysis

The qualitative analysis concerned the audiotapes of the interviews, working from verbatim transcriptions. A thematic analysis inspired from the methods described by Ragin [23], Miles and Huberman [24], Smith [25] and Apostolidis [26] was performed.

The first steps of this analysis were performed by two independent, blinded evaluators with different backgrounds (AD, a psychiatrist and FN, a methodologist). It consisted in 1/ immersion and familiarization with the data of the corpus (reading and re-reading of the interviews, notes), in order to obtain an exploratory coding of the first interviews and elements of interpretation, 2/ inventory and systematic identification of the emergent themes from the corpus analysed, and conceptual labelling of these themes to construct an analysis grid and 3/ application of the grid elaborated to detect relationships between the different prominent themes emerging from the verbatim. Agreement was reached in the course of different meetings. While we tried to have a very neutral approach by performing a content analysis that reflected, as best as possible, the verbatim collected, both reviewers were aware that some specific features of psychic change in pregnancy might be reflected in discourse, such as references to childhood, the early relationships of the pregnant women with their own mothers, clues about their attachment (secure/insecure with the expression of anxiety or fears) and/or maternal identity issues.

### Analysis phase three: integration of quantitative and qualitative analyses

After this first exploration of the qualitative data, the group to which each woman belonged was revealed to the evaluators and CO (the child psychiatrist who conducted the interviews) was allowed to participate in the qualitative analysis. This part of the study aimed to 1/ explore whether there were phenomenological differences between pregnant and non-pregnant women and 2/ identify the characteristics of women classified as pregnant or non-pregnant more often than would have occurred by chance. Agreement between evaluators was reached in the course of different meetings.

## Results

### Recruitment and characteristics of the women included

Fourteen pregnant women were selected, among whom 1/ four were not recorded (two declined due to a lack of time, one refused to be audiotaped and one did not attend the interview despite a earlier agreement) and 2/ three were excluded (two women made an explicit mention of their pregnant status during the interview and one was not able to produce 5 min of spontaneous speech). Seven matched controls were selected, recorded and analyzed. Table 2 provides the socio-demographic and clinical characteristics of the women included.

**Table 2:** Socio-demographic and clinical characteristics of women included

	Pregnant women	Non-pregnant women
Age	27 (25; 28)	28 (25; 31)
Education		
Bachelor’s degree	4 (57 %)	4 (57 %)
Master’s degree	3 (43 %)	3 (43 %)
Lifestyle		
Urban	4 (57 %)	4 (57 %)
Rural	3 (43 %)	3 (43 %)
Spielberger’s inventory		
< 35 : minimal anxiety	2 (29 %)	1 (14 %)
36–45 : slight anxiety	3 (42 %)	4 (58 %)
46–55 : moderate anxiety	2 (29 %)	1 (14 %)
> 55 : considerable anxiety	0	1 (14 %)
Significant medical illness	0 (0 %)	0 (0 %)

### Quantitative analysis

**Fig. 1:**
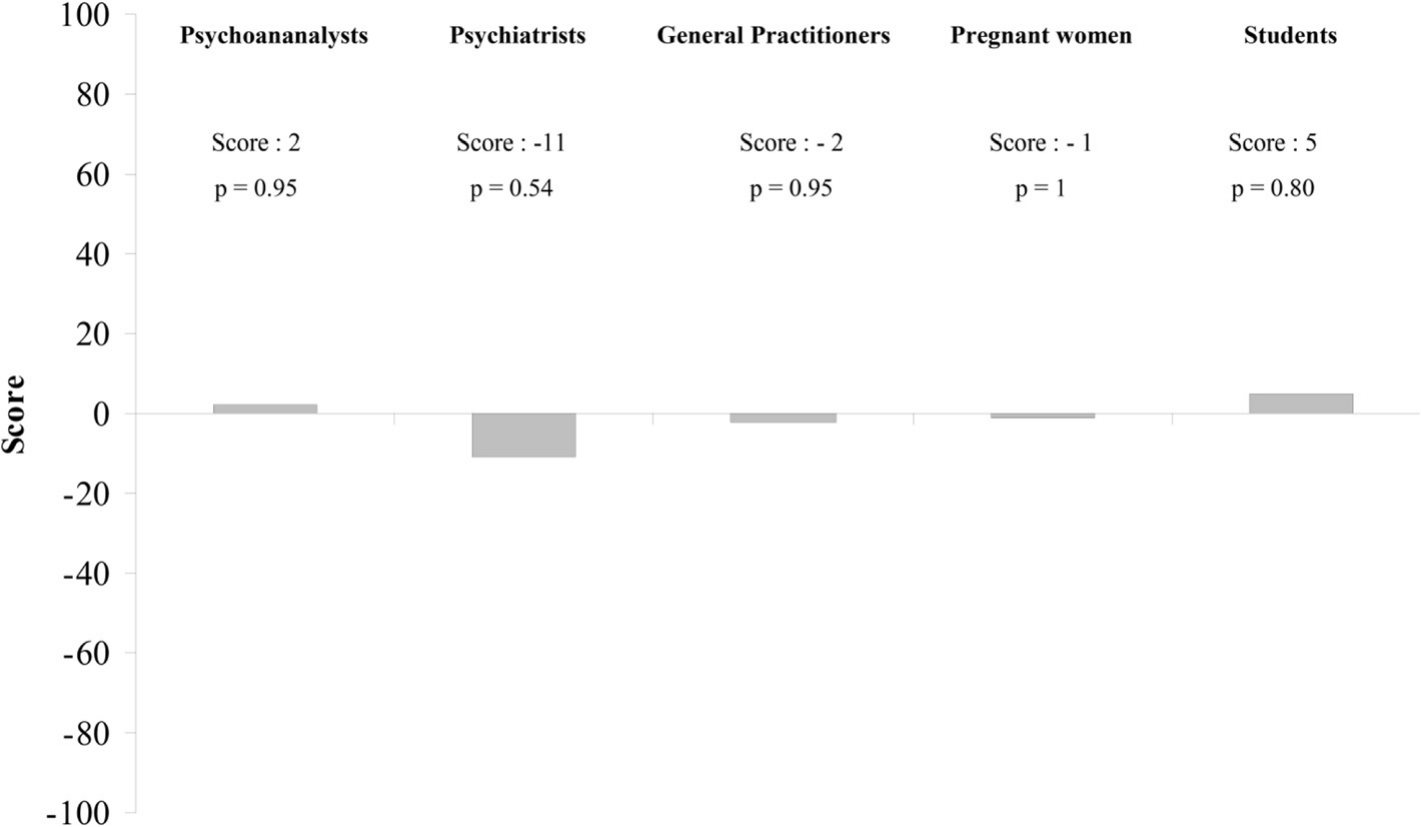
Scores obtained for the different groups of evaluators (with their *p-*values)

Individual results are presented in Table 3. No group of evaluators was able to correctly classify pregnant and non-pregnant women, with respectively *p* = 0, 95 for psychoanalysts, *p* = 0.54 for psychiatrists, *p* = 0.95 for general practitioners, *p* = 1 for medical students and *p* = 0.80 for pregnant women (Fig. 1).

**Table 3:** Raters’ scores according to how far they recognized or not whether healthy women (*N* = 14) were pregnant or not

	Psychoanalysts					Psychiatrists					General Practitionners					Pregnant women					Medical students					Total (woman)	Bilateral *p*-value (woman)
Rater	1	2	3	4	5	1	2	3	4	5	1	2	3	4	5	1	2	3	4	5	1	2	3	4	5		
Pregnant women																											
P1	1	2	2	1	−1	−2	−1	1	−2	−2	1	1	2	2	1	2	−2	−1	1	1	−1	−1	2	−1	2	8	0,34
P2	1	2	−1	−1	1	1	2	−2	2	2	−2	−2	2	−2	2	1	−2	−2	1	2	−1	1	2	1	2	10	0,23
P3	−2	−2	−1	−1	1	−2	2	−2	−2	2	−1	1	−2	−1	−2	−2	2	−2	−2	−1	1	−1	1	2	2	−12	0,14
P4	−2	−2	−1	1	−1	−2	−2	1	1	−2	−2	−2	−1	−1	−2	−2	2	−2	−1	−1	1	−1	−2	−1	−2	−26	**0,002**
P5	2	2	−1	1	1	2	−1	2	−2	2	1	1	2	1	−2	1	−2	−2	1	−1	−1	1	2	2	2	14	0,09
P6	−1	−2	−2	−1	1	2	−2	−2	−2	−2	1	−2	−1	−2	−2	1	2	2	2	−2	−1	1	−2	−1	−1	−16	**0,05**
P7	1	−2	1	1	−1	2	2	−1	2	−2	2	2	1	2	2	−1	2	2	2	−2	−1	1	−1	−1	−2	11	0,18
Non-pregnant women																											
NP1	2	2	2	1	−1	−2	2	2	−2	2	2	2	1	−2	1	2	2	−2	2	2	−1	−1	−2	2	−1	15	0,07
NP2	−1	−2	−2	−1	1	−2	−2	−1	1	−2	1	−2	1	−2	2	−1	2	−2	−1	−2	−1	1	−1	−2	−1	−19	**0,02**
NP3	2	−1	2	−1	1	2	−2	2	2	2	−1	2	2	−1	−2	2	−2	−1	2	1	1	1	2	−2	−1	12	0,14
NP4	1	2	−1	1	−1	2	2	−1	−1	−2	1	−2	−1	1	2	−1	2	1	1	−1	−1	1	1	1	2	9	0,28
NP5	−1	−1	−1	1	1	2	1	−1	−2	1	−1	−2	−1	2	−2	1	−2	1	1	−1	1	1	1	−1	2	0	1,00
NP6	2	2	−1	1	1	−2	−2	1	1	−2	−1	2	1	1	−1	−1	−2	−2	2	−1	−1	−1	2	1	2	2	0,85
NP7	−1	−1	−1	−1	−1	2	−2	−2	1	−2	2	1	−2	−2	−2	1	2	−1	−1	−1	−1	−1	−2	−1	1	−15	0,07
Total (raters)	4	−1	−5	2	2	3	−3	−3	−3	−5	3	0	4	−4	−5	3	4	−11	10	−7	−6	2	3	−1	7		

Scores are obtained as follows: +2 when the rater correctly answered yes or no according to whether or not each healthy woman was pregnant or not, +1 when they correctly answered probably yes or probably no, -1 when they incorrectly answered probably yes or probably no, and -2 when they incorrectly answered yes or no

Total scores are given 1/ in row for each rater and 2/ in line for each woman (with a bilateral *p*-value)

Bold data: statistically significant

Underline data: trend toward statistic significance

On the other hand, concerning each woman’s global score, statistically significant scores were obtained for two pregnant women. These two women were often rated as non-pregnant, more often than would have expected by chance (P4, *p* = 0.002; P6, *p* = 0.05). One non-pregnant women was considered as pregnant more often than would have been expected by chance (NP2, *p* = 0.02). Additionally, trends toward statistically significant scores were observed for one pregnant women who was generally correctly identified (P5, *p* = 0.09), one non-pregnant women who was generally correctly identified (NP1, *p* = 0.07) and one non-pregnant women who was generally wrongly identified (NP7, *p* = 0.07).

### Qualitative analysis

Data saturation was obtained during the analysis our corpus (i.e. for the last interviews included in the analysis, we did not obtain any new data). Four main themes were identified from the thematic analysis (Table 4): 1/ the link between dream and reality, 2/ the question of the other and otherness, 3/ the anxiety arising from the loss of self-control in dreams and ramblings and distinct coping profiles 4/ the notion of time and temporality. Each of these themes was divided into distinct subthemes presented in Table 4.

**Table 4:** Results of the qualitative analysis: thematic analysis

Themes	Subthemes	Explanation	Example
Link between dream and reality	On/off	Waking up suddenly without any residual element of the dream.	“Sometimes, I’m dreaming and I wake up suddenly…[…] with the feeling of stepping from one world to another”.
	Dream/reality entanglement	Feelings and perceptions during the day that may be linked to dreaming activities.	“Dreams can come back during the day… in a situation or when you’re reading something.” “When I dream about someone …and when I meet them during the day, it feels strange”
	Gradient	Sliding slowly from dreams to reality in the morning.	“Sometimes when I wake up I am still dreaming.” “Sometimes I start dreaming and then I’m in my patient’s room…wondering how I got there“ (woman working at night as a nurse).”
The Other and otherness	Self-understanding to understand others	Experiencing hypersensitivity to others’ feelings – the “emotional sponge”. (mopping up emotion)	“If my family or friends are not well, I’m not well either, I'll try to find out what’s wrong to help them.”
	Self/others entanglement	Guessing people’s feelings based on one’s own perceptions.	“I pay attention to others […] I can tell what people feel by listening to myself and my own feelings.”
Anxiety and coping	Death and disintegration (anxiety)	Fearing one’s own death or another’s death.	“I had this dream where my little sister was dead.”
	Identity (anxiety)	Feeling of no longer recognizing others and oneself.	“Sometimes, I dream about people I know but they don’t look like they should” “I dreamt I was a doll and I had two faces: my own face and the doll’s face”.
	Separation (anxiety)	Fear of drifting apart from those close .	“I dreamt that my mother came to visit me and suddenly [when she wanted to come in] my flat didn’t have doors anymore.”
	Verbalisation (coping)	Feeling the need to tell someone about the dream in the morning.	“Sometimes, my roommates tell me about their dreams”
	Reality appraisal (coping)	Checking the reality of some feelings or some memories experienced at night in real life.	“I dreamt my sister was dead, I woke up at 6 AM and phoned her to be sure she was OK even though I knew it was just a dream”
	Creative activity (coping)	Writing or producing art from dreams.	“I have a diary. I write up some dreams, telling myself that maybe, one day, I’ll publish it.”
	Rationalisation (coping)	Giving no meaning to the dreams.	“Some dreams have no meaning, they just couldn’t happen in real life so there’s no need to give them any credit.”
	Therapeutic function of dreams (coping)	Having the feeling that dreams lead to understanding what remains doubtful or unconscious during the day.	“It’s a way my brain is working at night, maybe to digest fears or, else pleasures.”
	Intellectualization through literature (coping)	Trying to use literature to understand dreams.	“I tried to understand my dreams with books, Freud for example.”
Temporality	Biography	Referring to personal life.	“I’m going to a seminary next week.” “About my professional project, I would like to stop working with old people […] maybe psychiatry […] definitely not in paediatrics.”
	Dreams	Referring to the time when dreams occur and to the connexions between dream content and the subject’s personal life.	“Some dreams keep on coming back over the years.” “I often dream in the morning, from 5 AM to 7 AM”
	Interview	Describing how they managed during the 5-minute interview	“5 min is a very long time.”

### Integration of qualitative and quantitative analyses

In line with the quantitative analysis, and despite some inter-individual nuances, all themes and sub-themes identified were common to all fourteen interviews and did not enable differentiation between the two groups.

The description of women classified as pregnant or non-pregnant more often than would have occurred by chance is given in Table 5. Two levels of understanding could be highlighted by this analysis. The first level is that some clues in the interviews could have influenced the evaluators’ choices. Fluent speech, with numerous references to childhood was particularly observed in women identified as pregnant. In contrast, discourse full of professional concerns or material themes was observed in women identified as non-pregnant. This trend was obvious for the “statistically significant” women and was more subtle for women for whom there was a trend toward statistical significance (Table 5). The second level of understanding is that the distinction between pregnant and non-pregnant women could have been confused by a psychological characteristic: their “desire for a child”. Indeed, it seems that P4, who was very often wrongly identified as non-pregnant, revealed that the current pregnancy was neither planned nor desired, whereas N2, who was often wrongly identified as pregnant, was questioning herself about her desire for children. Moreover, N7, often wrongly considered as pregnant, was planning to get married and to become a mother soon.

**Table 5:** Description of women classified as pregnant or non-pregnant more often than would have occurred by chance (significant *p*-value or trend toward statistical significance)

Women	Pregant or not pregant	Classified wrongly or rightly	Women’s characteristics (not audiotaped)	Interviewee characteristics (audiotaped)
Statistically significant				
P4	Pregnant	Wrongly	This 24-year-old woman said that the current pregnancy was neither planned nor desired. She also said that she had integrated this state and now was happy about it.	Speech pervaded by professional concerns, both current and future. She explicitly mentioned that she did not want to work with children.
P6	Pregnant	Wrongly	This 27-year-old woman mentioned no particular preoccupation with her pregnancy.	Speech pervaded by professional concerns. She said she was working at night.
NP2	Non-pregnant	Wrongly	This 34-year-old woman reported many questionings about motherhood and the desire for children. She was wondering whether she wanted to be a mother.	Fluent speech abounding in themes in connection with childhood.
Trend toward statistic significance				
P5	Pregnant	Rightly	This 29-year-old woman described a desired pregnancy. She was feeling peaceful about her pregnancy and described a change in her relationships (being more tolerant, with a better ability to communicate for example), which contrasted with her usual manner.	Fluent speech with numerous references to her family. She talked about her dreams using metaphors. She sometimes referred to her happiness.
NP1	Non-pregnant	Rightly	This 32-year-old woman was very comfortable with free association speech, she mentioned her personal analysis (stopped when interview time ran out).	Speech rooted in the present with loose associations. Talk about her roommate and the importance she attributed to her dog. She concluded, however with questionings about the desire for a child and what could motivate women to procreate.
NP7	Non-pregnant	Wrongly	This 26-year-old women was very excited about getting married in the coming weeks, she was also planning to be pregnant right after the wedding.	Fluent speech with numerous themes in connection with the childhood and anxiety. Evocation of her professional activity in connection with children.

## Discussion

### Summary of results

Neither the group of the psychoanalysts nor the other groups correctly classified pregnant and non pregnant women after listening to a 5-minute audiotaped spontaneous speech sample. In addition, the qualitative analysis of data suggests that no characteristic of the spontaneous discourse was specific of pregnant women. These findings go against the concept of “psychic transparency” which has been described as something that is patently obvious, characteristic of pregnancy and very easy to identify [6].

The concept of psychic transparency arose from the clinical case study method. The researcher bias [10] linked to it may constitute a possible explanation for our results. Indeed, in our experiment, the evaluators’ choices could have been biased by social representations of pregnancy. Typically, some women classified as pregnant by the evaluators presented fluent speech focused on childhood within a peaceful perception of their lives, whereas women classified as non-pregnant presented speech focused on their professional life and expectations about it. Our data suggest that these preconceptions were shared by all groups.

A second explanation is that the particular psychic state that might be observed during pregnancy is not specific to the state of pregnancy in women. Indeed, our data suggest that the “desire for a child” is a confounding factor which could possibly have been better detected by the different evaluators than pregnancy itself. This last result suggests the idea that the change presumed to occur during pregnancy could potentially occur earlier than the pregnancy itself, from the moment the desire for children takes shape in the minds of women.

### Limitations

Our experimental procedure was designed to detect large differences between accurate response and chance (high specificity and sensitivity). The five-minute audiotaped interviews may have been too short to detect a specific psychic state associated with pregnancy and the number of subjects/raters can be considered small from both the quantitative and qualitative points of view.

Nevertheless, 1/ psychic transparency has been described as something that is fairly obvious [6, 27] and 2/ the same study design has previously made it possible to show that psychoanalysts were able to recognize subjects who had a sibling with cancer during childhood, which was thought to be a non-obvious state [12], 3/ the number of subjects/raters was based on conservative hypotheses as regards type-one and type-two errors [13] and 4/ the two investigators who performed the qualitative analysis had the impression that it reached data saturation [9] since no new theme emerged from the last interviews. This last point should not be over-interpreted since it must be understood only as a shared subjective construct.

Efforts were made 1/ to use inclusion criteria selecting a very homogeneous sample and 2/ to match women for age, education level and lifestyle. Nonetheless, the matching did not concern personality traits, which could have influenced the results observed. Moreover, whereas the term “psychic transparency” refers to a personal change, our cross-sectional design did not enable comparison between the psychic state observed during pregnancy and the psychic state previous to pregnancy. Prospective studies are therefore required to tackle this issue.

Finally, the women were instructed not to mention directly or indirectly their state (pregnant or not). This directive could have limited their freedom of association. Moreover, for this reason, two audiotaped pregnant women were excluded because they made an explicit mention of their status during the interview. It is possible that, even if “psychic transparency” is described as reaching its maximum during the third trimester [6], it was difficult for women not to mention their condition, considering how close they were to delivery [17]. This could have resulted in a possible selection bias for women in the pregnant group (selecting those with better cognitive control). This merely illustrates the fact that any experimental context can have an impact on the results they generate, especially when very challenging concepts like those developed in the psychoanalytic framework are explored. And indeed, our experimental design produced a somewhat artificial situation and a research interview of this type (where the data is treated as a static object) is not entirely comparable with a psychoanalytic session (which is rather a dynamic and changing object). In line with this comment, the introductory probe sentence we used could be overly directive for speech to emerge freely and spontaneously. In addition, the intersubjective space of the transference/ countertransference encounter, unconscious desires and defenses is built up session after session and provides material for psychoanalytic understandings.

The negative results of the present study should be interpreted cautiously, in line with these very challenging limitations. We believe that the interest of our study is that it can stimulate debate about these concepts and their validity, and can suggest a suitable way to explore them. Even if “psychic transparency” does not seem to be obvious, our results do not make it possible to claim that a definitive answer has been reached.

### Perspectives

Because they combine the thoroughness of a hypothetico-deductive approach and the completeness of a constructivist approach, mixed methods [28] are perfectly suited to tackling questions in the field of experimental psychology, in particular in the context of psychodynamic psychopathology. These methods enable exploration of both the concept under study and the possible presence of a researcher bias.

In the particular context of our study, these methods showed that the phenomenon of “psychic transparency” was far from obvious and that the evaluators’ ability to differentiate pregnant and non-pregnant women was probably more dependent on their social representations than on a genuine state resulting from pregnancy. Indeed, social representations convey a positive picture of what a future mother should be. In advertisements, for example, or in magazines, the pregnant woman is often represented as relaxed, blossoming, serene, smiling, happy and not working. Many women do not subscribe to these preconceptions. The concept of “psychic transparency” could also entail a representation of what the psyche of pregnant women should be. But here again, the link with pregnancy is not optimal. In a more global perspective, it emphasises the fact that interpretations made by clinicians in day-to-day practice are constructions between their patients’ reality and their own. Within these constructions, social representations could be misleading. Helping the professionals working in the perinatal area to take these mechanisms and representations into consideration, helping them to get away from their own beliefs, whatever the specialty (psychiatrist, psychoanalyst, etc.), could influence their practice and contribute to the field of prevention.

## Conclusions

Overall, this experiment does not support the view that a specific state called psychic transparency enables obvious recognition of pregnant and non-pregnant women, even for psychoanalysts. More subtle changes need to be explored.

## Abbreviations

STAI, State-Trait Anxiety Inventory
